# Design of standalone wireless impedance matching (SWIM) system for RF coils in MRI

**DOI:** 10.1038/s41598-022-26143-9

**Published:** 2022-12-14

**Authors:** Sri Kirthi Kandala, Sung-Min Sohn

**Affiliations:** School of Biological and Health Systems Engineering, Arizona State University, Tempe, 85281 USA

**Keywords:** Biomedical engineering, Electrical and electronic engineering, Magnetic resonance imaging

## Abstract

The radio frequency (RF) power transfer efficiency of transmit coils and the signal-to-noise ratio (SNR) at the receive signal chain are directly dependent on the impedance matching condition presented by a loaded coil, tuned to the Larmor frequency. Sub-optimal impedance condition of receive coils significantly reduces coil sensitivity and image quality. In this study we propose a Standalone Wireless Impedance Matching (SWIM) system for RF coils to automatically compensate for the impedance mismatch caused by the loading effect at the target frequency. SWIM uses a built-in RF generator to produce a calibration signal, measure reflected power as feedback for loading change, and determine an optimal impedance. The matching network consists of a capacitor array with micro-electromechanical system (MEMS) RF switches to electronically cycle through different input impedance conditions. Along with automatic calibration, SWIM can also perform software detuning of RF receive coils. An Android mobile application was developed for real-time reflected power monitoring and controlling the SWIM system via Bluetooth. The SWIM system can automatically calibrate an RF coil in 3 s and the saline sample SNR was improved by 24% when compared to a loaded coil without retuning. Four different tomatoes were imaged to validate the performance of SWIM.

## Introduction

Magnetic Resonance Imaging (MRI) is a non-invasive technique that uses radio frequency (RF) coils to perturb the spin of a nucleus and acquire the nuclear magnetic resonance (NMR) signal, which is reconstructed into an image^1,2^. Ultra-high field (UHF) strengths (7 T and above) provide better image quality in terms of higher signal-to-noise ratio (SNR), spatial resolution and contrast^3–7^. As the field strength increases, the operating frequency of the RF coils increases in accordance with the Larmor equation as shown in Eq. (1)^8^. Where *f* is the frequency (MHz), γ is the gyromagnetic ratio (MHz/Tesla) and B_0_ is the static magnetic field strength (Tesla).

1 $$f= \gamma {B}_{0}$$

The gyromagnetic ratio (γ) of ^1^H (proton imaging) is 42.6 MHz T^−1^. For field strengths of 1.5 T, 3 T, and 7 T the frequency of operation is 63.9 MHz, 127.8 MHz, and 298.2 MHz respectively. At 7 T, the ideal frequency of operation is 298.2 MHz. But the scanners tend to have slightly varied static field strength after installation for various reasons. Due to this each scanner has a specific frequency of operation for proton imaging. RF coils function as antennas for MRI scanners, generating alternating local magnetic fields (B_1_) perpendicular to the static field (B_0_) generated by the main magnet^9^. RF coils can be categorized as transmit coils, receive coils, and transceiver coils. Volume coils with designs like the birdcage coil^10,11^ and the transverse-electromagnetic coil (TEM)^12,13^ are usually used as transmit coils due to their uniform field distribution. Surface coils, such as loop coils or arrays of loops, are placed closely to the sample to detect the MR signal due to their high sensitivity^9,14,15^. An RF front end in MRI uses the standard concepts of conjugate and noise matching for power delivery efficiency and lower noise performance, respectively. Power amplifiers and low-noise amplifiers in MRI are separated from the coils with long coaxial cables, thus most coils are conjugate-matched to 50 Ω. Different samples in a coil change the impedance conditions and this loading effect can be measured by the amount of power reflected to the source. The reflected power measurement is used by a microcontroller to compensate for the impedance change of a coil. Automatic or adaptive impedance matching has been extensively explored in the wireless communication domain for mobile phones^16–20^, RFID^21,22^, and in wireless power harvesting^23,24^. Due to the use of lower frequencies in MRI, most of the hardware is not as compact as in the aforementioned technologies. All components of the MRI system must be non-magnetic; this factor restricts the use of ferrite isolators for measuring the reflected power. Therefore, automatic impedance matching systems in MRI are both expensive and bulky compared to their 5G or RFID counterparts.

Previous investigations in the field of MRI have shown promising development towards an automatic tuning and matching system for RF coils. The necessity for automatic tuning and matching was identified in MRI’s infancy^25,26^. Varactors were used to change the capacitance value to compensate for impedance mismatch^26,27^. Both magnitude and phase measurement compensation were also introduced in the past by using a ‘zero-crossing’ technique to find the optimal impedance matching state^28^. A PIN diode-based tuning and matching system was developed for an 8 channel transceive TEM coil using field-programmable-gate-arrays (FPGA)^29^. By simply retuning the coil, it was observed to restore a majority of the SNR. Frequency detection technique was used in stretchable liquid metal coils to compensate for frequency shift during stretching^30^. Recently, a low-cost automatic system with an Arduino and a phase-locked-loop (PLL) was developed to tune and match an RF coil over a large range of field strengths from 1 to 23 T^31^. Nonetheless, the idea of reading reflected power from the load to tune and match has remained the same for most part^32–35^. A preliminary study of this work using Arduino Nano and Xbee wireless communication has been reported^36^. An unaddressed issue with existing systems is the lack of real-time monitoring and control by the user. Current systems require synchronization with the MRI console using hardware connection and are designed for specific coils rather than general-purpose system. Also, the systems do not include any type of detuning feature for an RF receive coil. Detuning circuits are essential for a receive coil and most coils are designed with PIN diode or MOSFET detuning circuits. Such coil designs can potentially generate interference causing B_1_ field non-uniformity and the required bias for the active devices causes additional noise^37,38^. To mitigate these concerns, a novel software detuning technique was introduced. With these factors in mind, we have delved further into improving the hardware and software components of existing automatic impedance matching systems for MRI.

**Figure 1:**
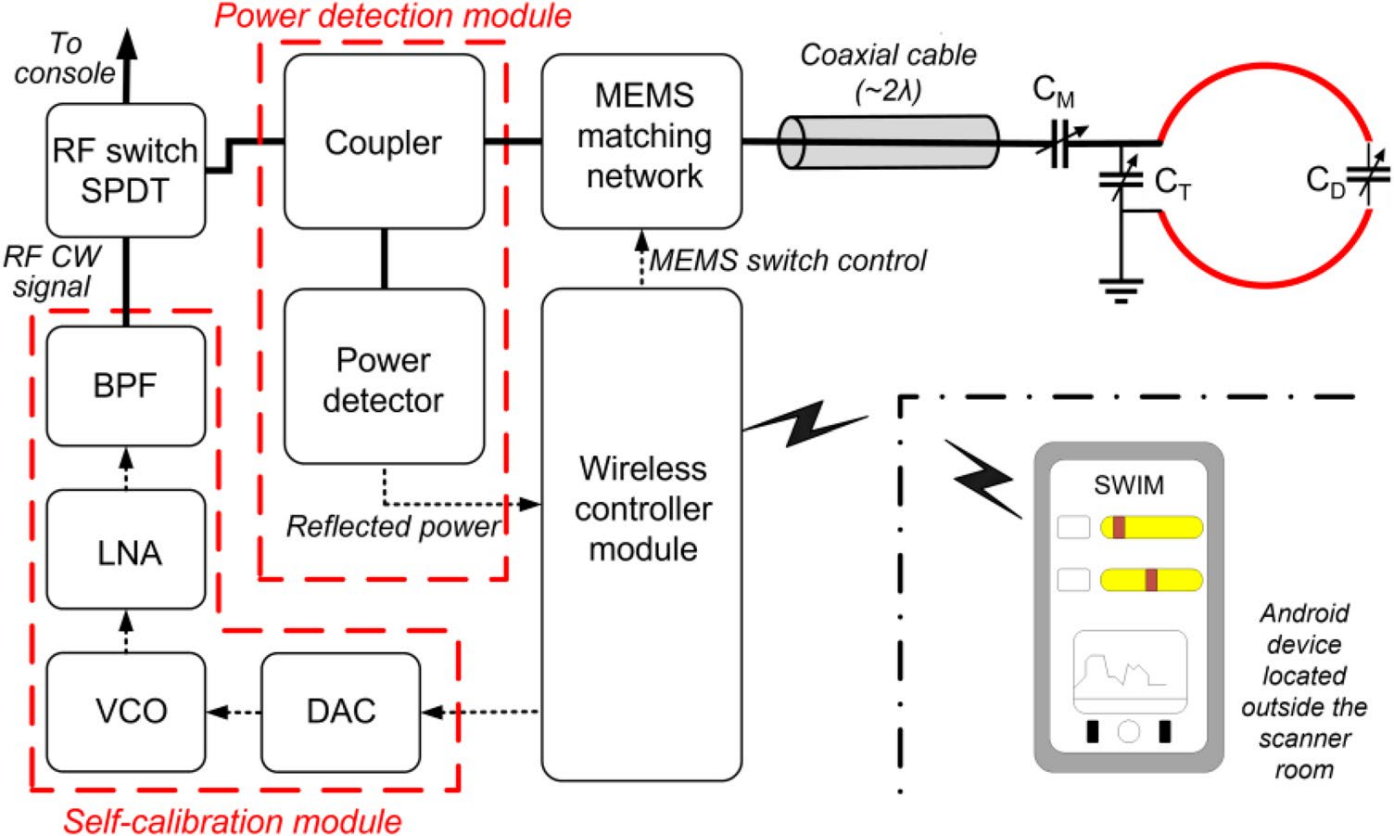
System level block diagram of the proposed SWIM system.

In this paper we present a standalone wireless impedance matching system using micro-electromechanical system (MEMS) RF switches to change the impedance of the RF coil as shown in the Fig. 1 system level block diagram. SWIM has a built-in RF continuous wave (CW) signal generation for self-calibration, which makes it completely standalone. SWIM uses the magnitude of reflected power from the coil as feedback to compensate for the impedance mismatch. We also developed a pseudo-manual control of the tuning and matching network to adjust the impedance, irrespective of the automatic feature. Apart from automatic impedance matching function, this pseudo-manual control allows the user to fine-tune the impedance condition after the automatic function. The complete system is wireless and controlled by an in-house developed Android mobile application via Bluetooth.

## Methods

This section discusses in detail the design of RF coil and phantom used in this project, each individual module of the SWIM system, and the android-based application developed to control the SWIM system. The SWIM system consists of one RF input, one RF output, and a DC power supply port. The RF coil is connected to the output of the SWIM system, the RF input is connected to the MRI console, and a 9 V is supplied at the DC port. The android application is loaded on a smartphone/tablet and connected to the wireless controller of the SWIM system via Bluetooth.

### RF coil and phantom design

**Figure 2:**
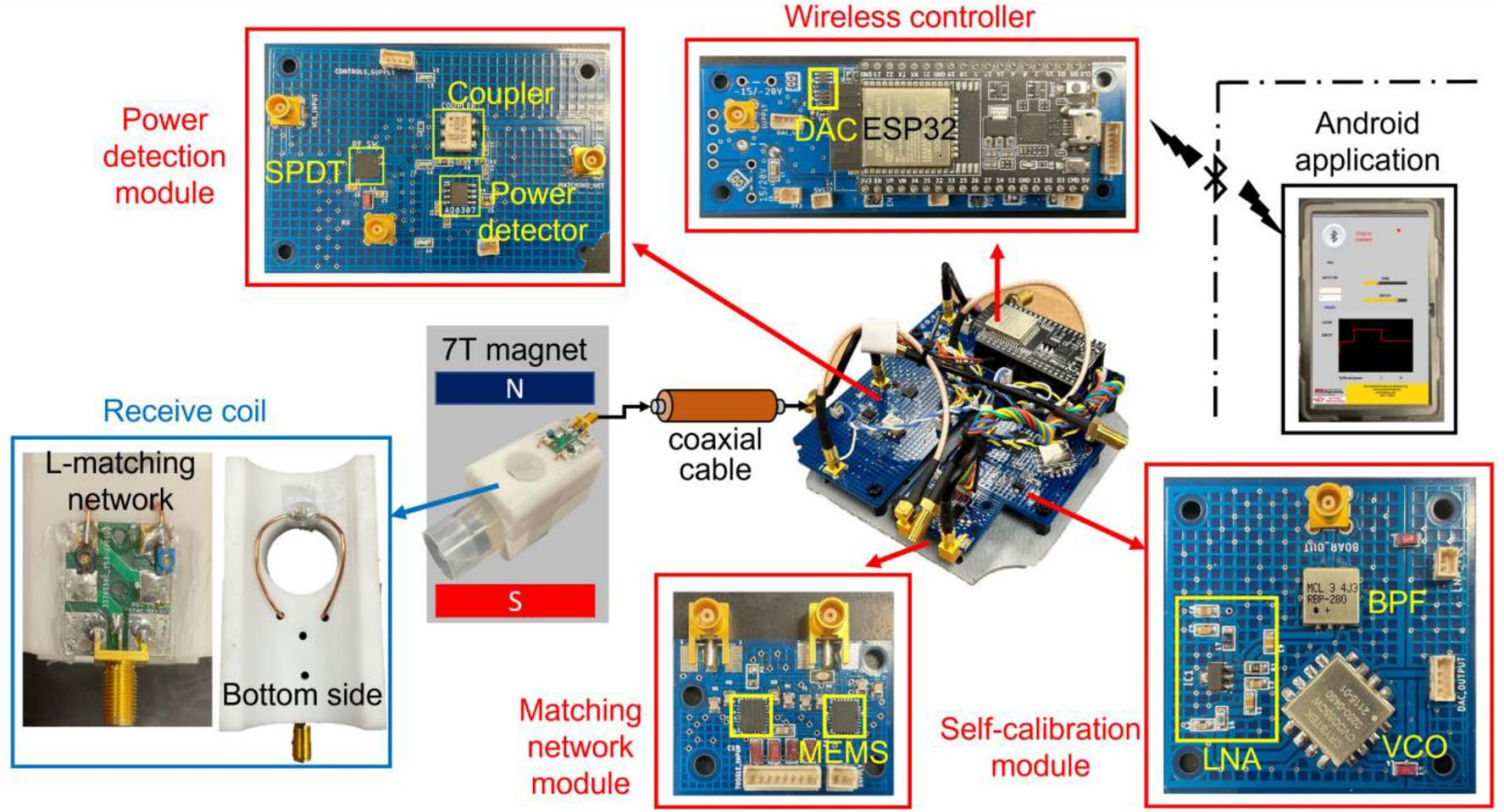
SWIM system setup with fabricated receive coil and 2 m coaxial cable for remote automatic tuning and matching. (Inset) detailed images of individual printed circuit board modules and receive coil design.

A single loop RF coil was designed and fabricated. A housing structure was resin printed to securely hold the phantom close to the coil. The loop was chosen to have a diameter of 28 mm with a single distributive capacitor (1111C Non-magnetic capacitors, Passive Plus Inc, USA) to ensure uniform current distribution. An L-matching network with shunt-tune, series-match capacitors (SG9128, Sprague Goodman, USA) was chosen as shown in Fig. 2. To verify the performance of the coil, a centrifuge tube of 30 mm diameter was chosen as the phantom to represent a rat head. The tube was filled with distilled water, CuSO_4_ (1 g/L), and Agar (10 g\L). The phantom is Agar-based to mimic brain tissue properties and the combination of copper ions and Agar concentration affects the proton relaxation time constants. It was observed that copper ions predominantly control the T_2_ whereas, the T_1_ depends more on agarose concentration^39^. Phantom images were used to observe the change in the B_1_ field strength of the surface coil before and after the automatic calibration with the SWIM system.

### The SWIM system

Manual tuning and matching of RF coils requires substantial time. Most coil systems present themselves with a sub-optimal matching condition after loading the sample. The loading effect creates an impedance mismatch and loss of signal that cannot be recovered by a pre-amplifier, thus deteriorating the SNR. The SWIM system is a fast, efficient, and cost-effective solution to impedance match any small animal 7 T receive coils. It is an appropriate combination of automation and user input to obtain desired results. The concept behind the SWIM system is to compensate for the impedance mismatch caused by loading effect of a sample by cycling through multiple capacitor combinations and choosing the optimal impedance based on a least reflected power measurement. This system is completely standalone and automatic since it generates its own CW signal by employing a voltage-controlled oscillator (VCO) for the process and does not depend on RF gating signal from the console. An external gating signal from the console is used as an input to the system for software detuning of the receive coil during the transmit cycle and not to synchronize the system with console for automatic impedance calibration. The RF coil developed does not use any independent circuitry to detune, instead, software detuning uses one of the 256 combinations as a detuned state. This proposed system can be divided into modules: (a) Self-calibration module, (b) Power measurement module, (c) MEMS-based matching network module, (d) Wireless controller module. The self-calibration module is connected to one port of the Single Pole Double Throw (SPDT) switch and the other port is connected to the MRI console. The coupler from the power measurement module is connected to the common RF port of the SPDT switch. The output of the coupler is connected to the MEMS matching network module and lastly the RF coil is connected to the output of the matching network as shown in block diagram Fig. 1. The wireless controller module connects to all 3 remaining modules to communicate and get feedback. Each of the modules is explained in detail in the following sub-sections.

#### Self-calibration module

This module consists of a VCO (CVC055CW-250-450, Crystek Crystal Corp., USA) which can generate a CW RF signal with a power of − 3 dBm. Two digital-to-analog converters (DAC) (MCP4725, Microchip Technology, USA) were used to adjust necessary voltages for the VCO. The microcontroller used inter-integrated circuit (I^2^C) serial protocol to interact with the DACs, which set up the supply and tuning voltages for the VCO. The RF signal was then followed by an E-pHEMT (Enhancement mode High-Electron-Mobility-Transistor) low noise amplifier (PHA-13LN + , Minicircuits, USA) to amplify the signal by 20 dB. A narrow band pass filter, designed in-house, was used as the next stage to filter out low frequency noise and higher harmonics from the VCO. The lumped element filter was designed to have a 54 MHz bandwidth at center frequency of 300 MHz. An SPDT CMOS RF switch (HSW2-272VHDR+, Minicircuits, USA) was used to switch the receive coil between MRI console (connecting to the receive chain LNA) and self-calibration module. RF common (RFC) port is connected to a bidirectional coupler, RF1 (port1) is connected to the MRI console and RF2 (port2) is connected to the self-calibration module. This switch offers high linearity with + 85 dBm 3rd order intercept point, low insertion loss, and internal CMOS driver, making it a perfect fit for low power impedance matching system. Insertion loss and isolation data of the switch were characterized at 300 MHz as 0.16 dB (RFC-RF1), 0.27 dB (RFC-RF2), and 47 dB (RFC-RF1/RFC-RF2) respectively. After the SWIM function, the switch will toggle to a slightly lower insertion loss path and the input impedance presented to the console can be fine-tuned wirelessly with the pseudo-manual function, via the mobile application.

#### Power measurement module

A bi-directional coupler (ADCB-20-82+, Minicircuits, USA) is used to tap the reflected power. This device has a low measured insertion loss of 0.2 dB, 20 dB of coupling at both ports, and a directivity of 30 dB at 300 MHz. By terminating the forward coupling port with a 50 Ohm resistor, an accurate reflected power measurement can be conducted. Reflected power was measured using a logarithmic amplifier-based RF power detector (AD8307, Analog Devices, USA). The amplifier uses a progressive compression technique with 6 amplification stages. A narrow-band input matching at the frequency of interest also helps in better signal sensitivity along with a certain amount of frequency selectivity. As the output of the coupler is unbalanced, using unequal capacitor values in the input matching network provides a balanced differential drive at both the input ports of AD8307. The OFFSET feature allowed us to change the intercept point and further extend the dynamic range of the power detector. The lower range is largely limited by the thermal noise floor. Taking these considerations into account, the external matching circuitry was designed to provide the necessary dynamic range of 90 dB at 300 MHz. The lumped element values C_1_—4 pF, C_2_—3.7 pF, and L_M_—120 nH were calculated using ADS simulation using the touchstone (.s2p) file of the device from the manufacturer. The power detector has a slope of 25 mV per dB. The analog output was fed to a 12-bit Analog-to-Digital (ADC) converter which was integrated in the microcontroller.

#### MEMS-based matching network module

**Figure 3:**
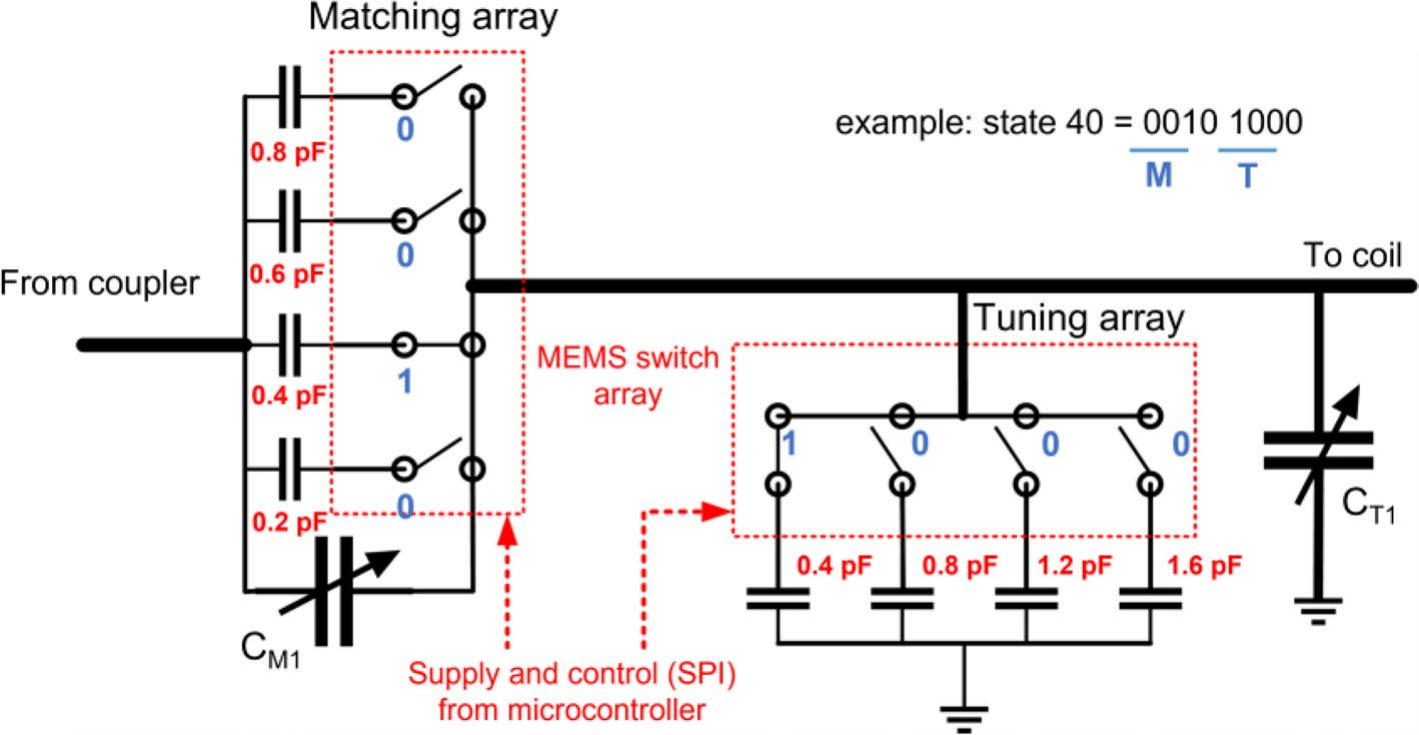
Matching network array design with individual fixed capacitor values and MEMS switches displaying one of the 256 combinations as an example to show individual MEMS switch configuration, where 0-switch off and 1-switch on.

Space inside a preclinical scanner is very limited and it was difficult to place the SWIM system inside the bore close to the RF coil without using a long coaxial cable. Therefore, we used a remote matching technique with two matching network boards^40–43^. An L-matching network with two trimmer capacitors (SG9128, Sprague Goodman, USA) close to the coil followed by a 2 m long coax cable extending out of the bore. We then connected the SWIM system outside the bore which includes a secondary L-matching network with two Single Pole 4 Throw (ADGM1304, SP4T, Analog Devices, USA) MEMS RF switches to create a capacitor array bank as shown in Fig. 3. This switch was chosen because of high linearity and low C_OFF_ and R_ON_ for each port. The insertion loss and isolation of any one of the 4 ports (RFx) to the common port (RFC) at 300 MHz were measured to be 0.16 dB and 47 dB respectively. The switch also measured a crosstalk isolation of 45 dB between two ports. In shunt configuration, RFC was connected to the trimmer capacitor and each of the 4 switches were connected with a fixed capacitor (0505C Non-magnetic capacitors, Passive Plus Inc, USA) to ground. Similarly, in series arrangement, 4 fixed capacitors were connected to form a capacitor array. We also used a 10 MΩ resistor in shunt with each fixed capacitor to avoid floating capacitance during the off state of the switch. Each of the 4 throws can be individually controlled using Serial Peripheral Interface (SPI) in a multiplexer (MUX) style to create 256 combinations. The on time of the MEMS switch was 75 μs, thus making the quickest time to cycle through all combinations without delays was approximately 20 ms. But a 5 ms delay between state transitions was introduced to account for RF settling time which makes a 3 s total run time for automatic tuning and matching sequence. The eight fixed capacitor values were picked by loading various sample sizes into the coil to understand the scope of impedance mismatch based on sample size. This method ensured that the SWIM system was not limited by capacitor values for a large variety of sample sizes.

#### Wireless controller module

The wireless microcontroller (ESP32-WROOM-32D, Espressif Systems, China) used in the SWIM system is a powerful, cost-effective, and easy to use module with generic Wi-Fi, Bluetooth, and Bluetooth Low Energy (BLE) capabilities. We used the built-in 12-bit ADC of the ESP32 to read the reflected power from the power detector and map it to each of the 256 states of the matching network array. SPI protocol is used to control the MEMS switch instead of digital pins as serial communication is robust and makes it easier to customize the system by adding more MEMS switches in the future. An array of digital pins is connected to power detector and power supply regulators to enable and disable these devices according to need. This reduces additional power supply noise and reduces power consumption of the SWIM system during imaging sequence as these devices are turned off. An Android mobile application can be paired with the Bluetooth feature to create a user interface to interact with the microcontroller.

### Android based application

**Figure 4:**
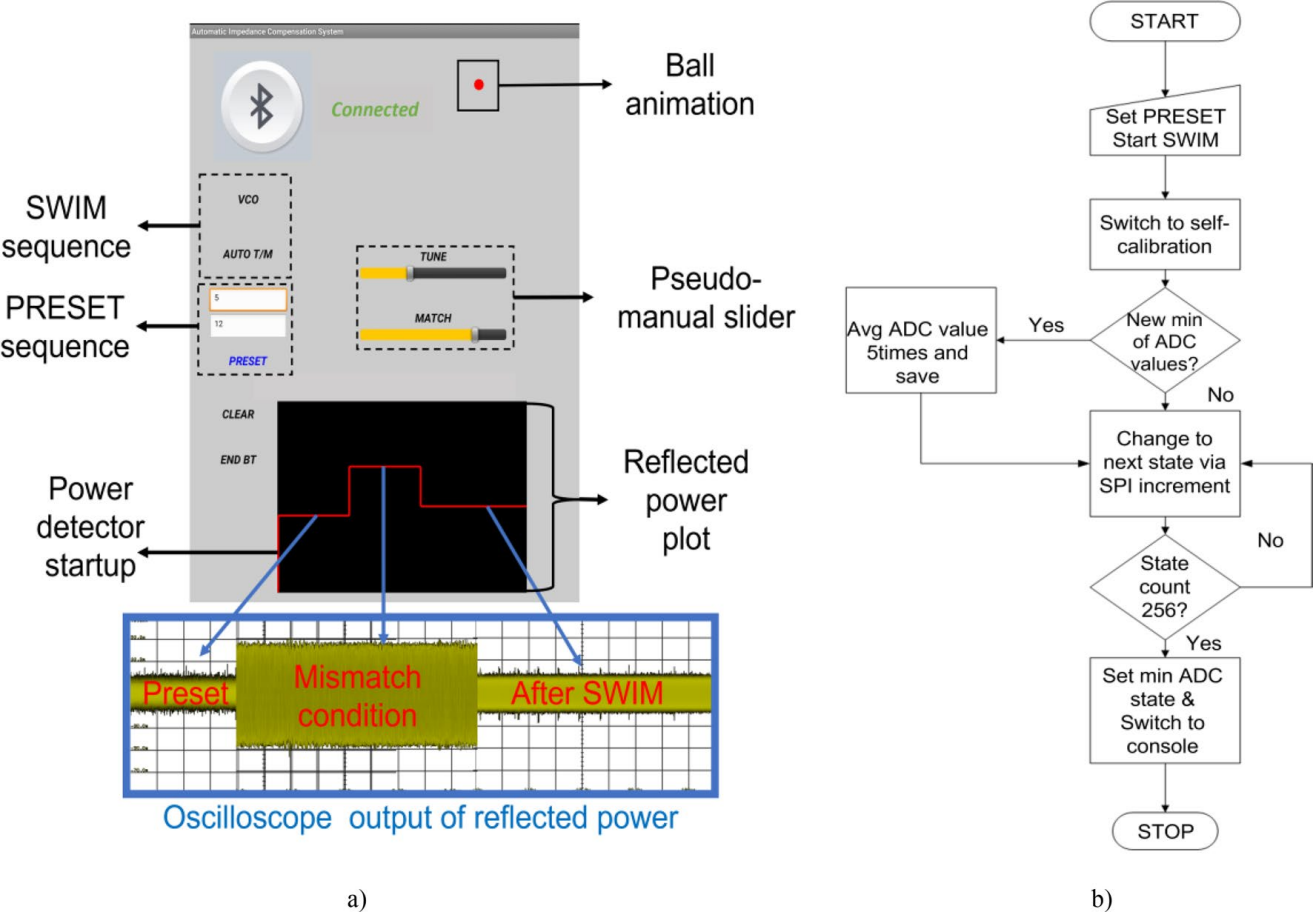
(**a**) Mobile application with real-time reflected power display (Inset) Oscilloscope display of the reflected power showing the three main stages of the operation (**b**) SWIM system algorithm describing its auto-calibration.

We have developed an Android mobile application using MIT App Inventor as shown in Fig. 4a. After pairing the SWIM system to the mobile device, an ESP32 module can be connected individually with a unique MAC address. Up to eight individual SWIM systems can be connected to the Android application and controlled independently, thus allowing the user to scale this design to accommodate up to eight channel RF coils. Two buttons were designed to independently control the self-calibration module and AUTO T/M feature. Two input dialog boxes were placed to set the remote tuning and matching separately based on the coil parameters, known as the PRESET condition. PRESET condition refers to decimal state of individual MEMS switches. Sixteen states each for tuning and matching, respectively. PRESET condition gives the user the ability to preset the SWIM system into certain impedance condition, before automatic calibration, based on coil design and loading effect. This will largely impact the ability to tune and match a sample. Similarly, two slider bars were designed to provide wireless pseudo-manual control of tuning and matching capacitor array independently and irrespective of the auto-calibration function. After the automatic function, these slider bars can also be used to fine-tune the impedance condition. They also provide an easy wireless adjustment of impedance condition between scans to account for sample movement. Finally, we also included a ball animation, which stops moving after the automatic sequence completes, as a notification. Auto-calibration sequence is shown as a flowchart in Fig. 4b. Once a receive coil is connected to the SWIM system with a preset condition for the MEMS switches, an initial reading of reflected power is displayed for preset state and plotted on the black canvas as well. This is followed by loading of the sample and impedance matching of the transmit coil. To match the receive coil the main RF path was switched to the self-calibration system and CW signal was enabled. When the AUTO T/M button is pressed, the system cycles through all the possible combinations and plots the reflected power at the end of the automatic function. At the end, the system locks on to the least reflected power state. We confirmed the optimal state by manually changing states and checking the reflected power. Next, the self-calibration system is turned off and the main RF path is toggled back to the console receive chain, ready for imaging. After being satisfied with the impedance of the coil, Bluetooth was disconnected, and the microcontroller was placed in deep sleep to avoid adding any electronic noise to the image.

## Results

### Circuit simulations

**Figure 5:**
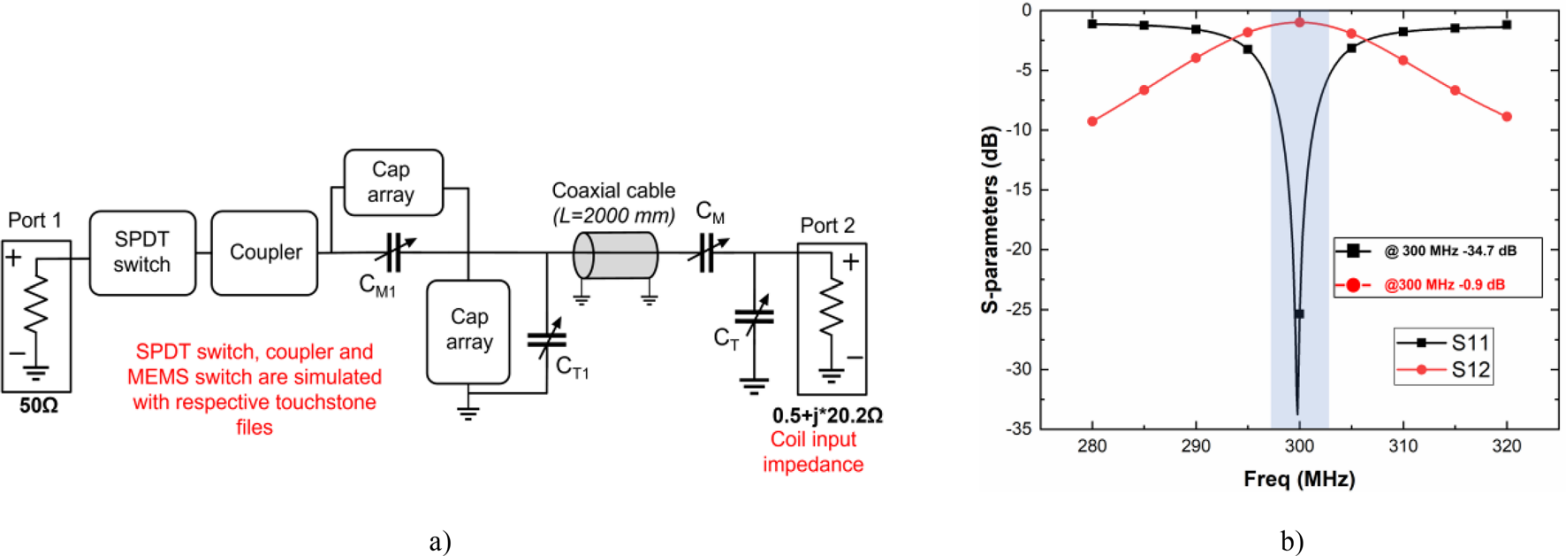
(**a**) Matching network simulation setup with 2 m coaxial cable to simulate remote tuning and matching and (**b**) S-parameter plot of the SWIM system modeled in ADS.

To understand the impact of a long coaxial cable (~ 1λ to ~ 2λ) and the SWIM system on the impedance matching of the coil we used PathWave Advanced Design System (ADS, Keysight) to simulate each block with respective touchstone files. A copper coil of 28 mm diameter was designed in ADS layout, converted to a symbol, and used to calculate the impedance of the coil at 300 MHz. Primarily an L-matching network was designed with two capacitors at the coil end. Then a long coaxial cable (~ 2 m) was modeled to understand the effect on impedance. Next, all the components in the SWIM system are modeled using touchstone files from the manufacturers as shown in Fig. 5a. The SWIM system insertion loss of 0.9 dB was observed at the center frequency as shown in Fig. 5b.

### Electromagnetic simulations in HFSS

**Figure 6:**
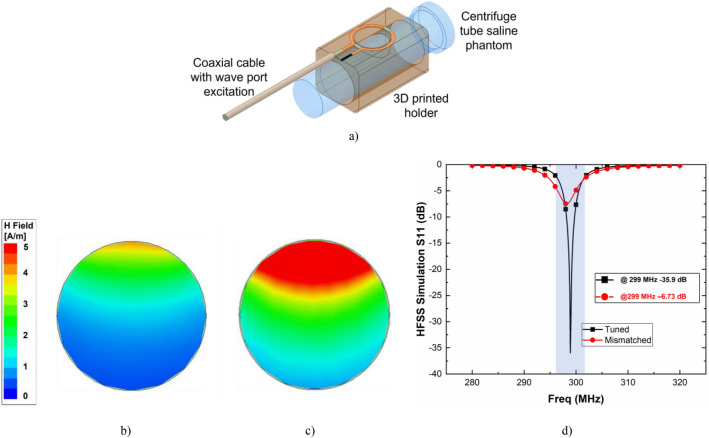
(**a**) Simulation setup in HFSS with 3D print housing and tube phantom (*Ansys, 3D High Frequency Simulation Software, Release 21.1,* https://www.ansys.com/products/electronics/ansys-hfss). Axial view of H-field inside the tube volume (**b**) mismatched condition due to loading and (**c**) retuned condition after loading, and (**d**) S11 plot of the HFSS simulation results for tuned and mismatched condition.

A loop coil was modeled along with its 3D-printed housing as shown in Fig. 6a simulation setup (*Ansys®, HFSS, Release 21.1)*. A 50 mL centrifuge tube modeled using polyethylene and filled with saline solution (permittivity, ε: 53 F m^−1^, conductivity, σ: 0.55 S m^−1^) and was placed 2 mm away from the coil to view the local magnetic (H)-field distribution in the region of interest (ROI). A perfectly matched layer (PML) was defined as the simulation boundary condition. When the sample was loaded, the impedance presented by the receive coil was changed. Initially, the tube was filled with air and the coil was tuned and matched at 299 MHz. Next, the (H)-field was plotted inside the volume as shown in Fig. 6b. Finally, the coil was retuned and matched with saline phantom at 299 MHz and the (H)-field recorded is shown in Fig. 6c. In Fig. 6d. the effect of impedance mismatch due to loading condition is shown in terms of s-parameters. Here, HFSS simulations show that S_11_ has reduced from − 35.9 to − 6.73 dB at 299 MHz after loading the sample.

### Measured results

**Figure 7:**
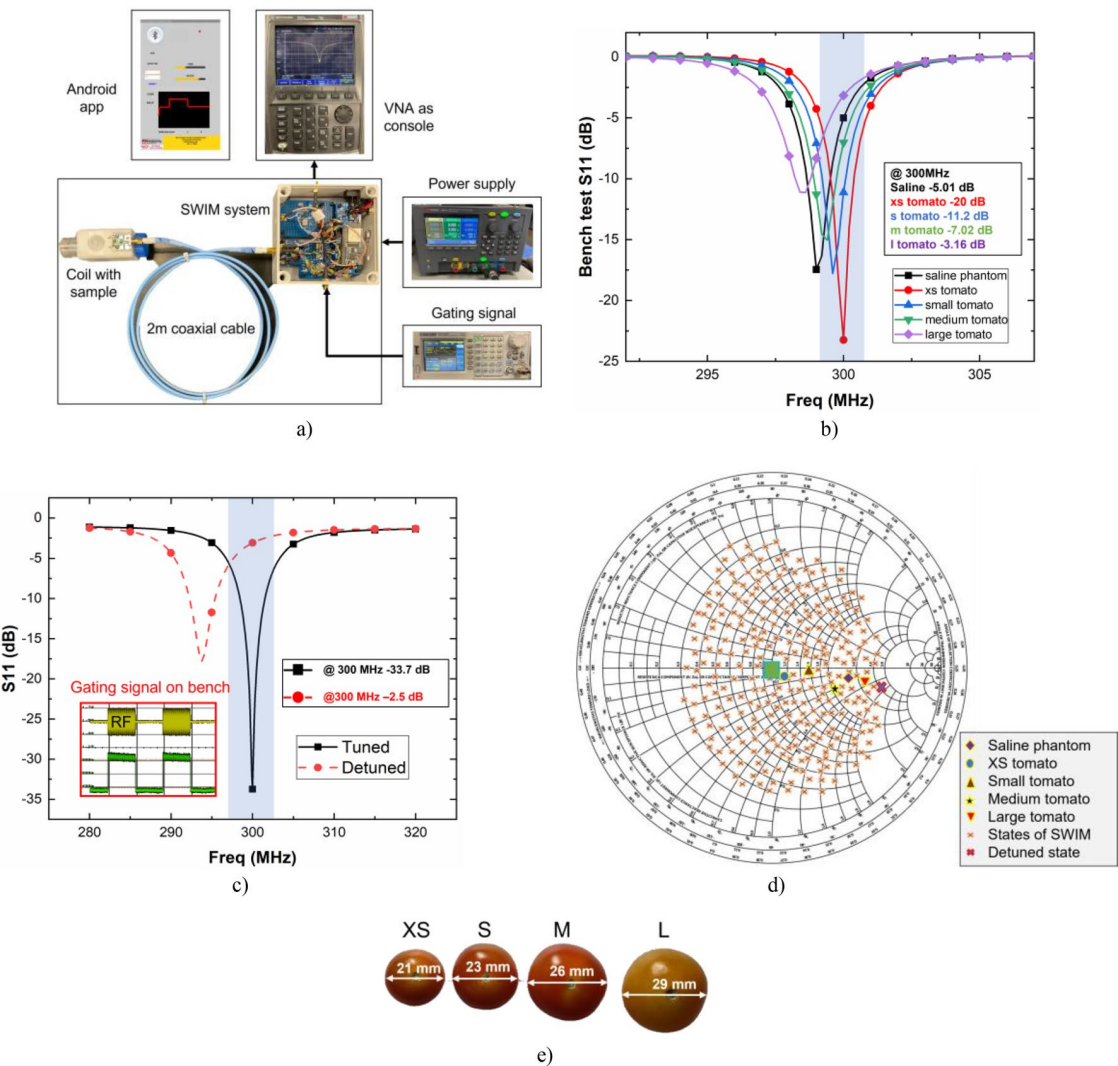
(**a**) Experiment setup to characterize SWIM system performance on the bench, (**b**) measured S11 data showing loading condition of various samples, (**c**) software detuning with (inset) external gating signal generated on bench, (**d**) impedance mismatch caused by different sized samples and complete tuning range of the SWIM system with 256 states, and (**e**) picture of different size tomatoes used for bench test and imaging.

Bench test measurements were collected using saline phantom. A two-port VNA (FieldFox N9923A, Keysight Technologies, USA) was used to gather the S-parameters. Tests were conducted both with and without the SWIM system to understand the impact of SWIM on the coil Q factor. Individual modules of the system were also tested to fine tune the system performance towards a more general-purpose SWIM design. Figure 7a shows the bench test setup where the network analyzer replicated an MRI console and was connected to the SPDT switch of the SWIM system. A 2 m coaxial cable was used to connect the fabricated receive coil to the SWIM system (cable is wound to capture a neat picture). A 9 V DC supply is connected to the SWIM system but a rechargeable non-magnetic battery with adequate current drive can replace the bench power supply for more portability. The impedance mismatch caused by loading various samples can be seen in Fig. 7b. As the sample size increases, the frequency decreases. The Q-factor of the simulation and bench tests were calculated using the following Eq. (2). Where, fc is the frequency of interest and BW is the 3 dB bandwidth of the device under test. Loaded Q and unloaded help to understand the coil sensitivity. Q_Unloaded_ and Q_Loaded_ of the coil and the SWIM system combined were calculated from bench data to be 146 and 48 respectively. Similarly, the loaded Q and unloaded Q values from simulation were computed to be 186 and 59 in that order. The Q_ratio_ of the coil must be greater than 2. A coil with ratio greater than 2 lies in the sample noise dominant regime. When the sample noise (R_S_) dominates, the reduction in coil noise (R_C_) only improves the SNR by a small quantity. Therefore, a Q_ratio_ larger than two means the coil is close to its peak performance. Due to higher sensitivity the simulation shows greater mismatch from loading the sample compared to the bench test. When the S-parameters are measured on the bench with SWIM connected to the coil, the response has a slightly lower Q factor. This decrease in Q can be corresponded to the 1.2 dB insertion loss of the components of SWIM.

2 $$Q=\frac{{f}_{C}}{{BW}_{3dB}}= \frac{\omega L}{R}$$

3 $$\frac{{Q}_{unloaded}}{{Q}_{loaded}}=1+\frac{{R}_{S}}{{R}_{C}}$$

MR consoles generate a transistor-transistor-logic signal which can be active-high or active-low based on the manufacturer and the SWIM programming has to be adjusted according to the scanner. An external RF gating signal (active-high) was generated using a pulse generator (SDG1025, Siglent Technologies, CHINA) to imitate the RF gating signal from the console as shown in (inset) Fig. 7c. This signal was then fed to the SWIM system via a logic level-shifter to a digital pin of the microcontroller to activate the software detuning feature. When the software detuning is triggered, it reduces the S_11_ from − 33.7 to − 2.5 dB at 300 MHz as shown in Fig. 7c thus effectively detuning the coil during transmit RF pulse. This shifted resonance ensures that there is little to no current induced in the receive coil at the center frequency during that transmit pulse, thus protecting the components of the receive chain. Extensive bench tests with different sample sizes were carried out to carefully determine the capacitor values of the matching network array to create a general-purpose detuning system. The range of the SWIM system is shown in Fig. 7d as each input impedance point is plotted on the Smith chart (orange x-cross). A total of 256 impedance states with one of them as the detuned state (blue x-cross). Each sample used in the bench test, as shown in Fig. 7e, creates an impedance mismatch based on sample size which is referred to as loading effect. If the mismatched impedance point lies within the tuning range of the SWIM system, it is possible to retune the RF coil with an input impedance in the vicinity of the green-square. The trade-off for the SWIM system is between the tuning range of the system and the resolution of the points within the tuning range. One solution is to increase the number states of the SWIM system at the expense of time take to tune and match an RF coil.

### MR images

All the MR imaging studies were conducted at Barrow Neurological Institute—Arizona State University (BNI-ASU), Center for Preclinical Imaging, using a 7 T small-animal, 30-cm horizontal-bore magnet and BioSpec Avance III spectrometer (Bruker, Billerica, MA) with a 116-mm high-power gradient set (600 mT/m). Fast-Low-Angle-Shot (FLASH) Sequence with a repetition time (TR) of 350 ms, echo time (TE) of 5.4 ms, and a flip angle (α) of 20 degrees was used for MR imaging experiments. A field of view (FOV) of 40 × 40 mm and 256 × 256 matrix leading to an in-plane resolution of 156 × 156 μm, in addition 9 slices were acquired along the sample with a slice thickness of 1 mm. A Bruker Linear Birdcage coil was used as the transmit coil. The peak power used for the FLASH sequence was 700 W. A rat bed of 72 mm diameter was used to hold the receive coil housing. Sample was then placed inside the receive coil housing and fed into the scanner.

**Figure 8:**
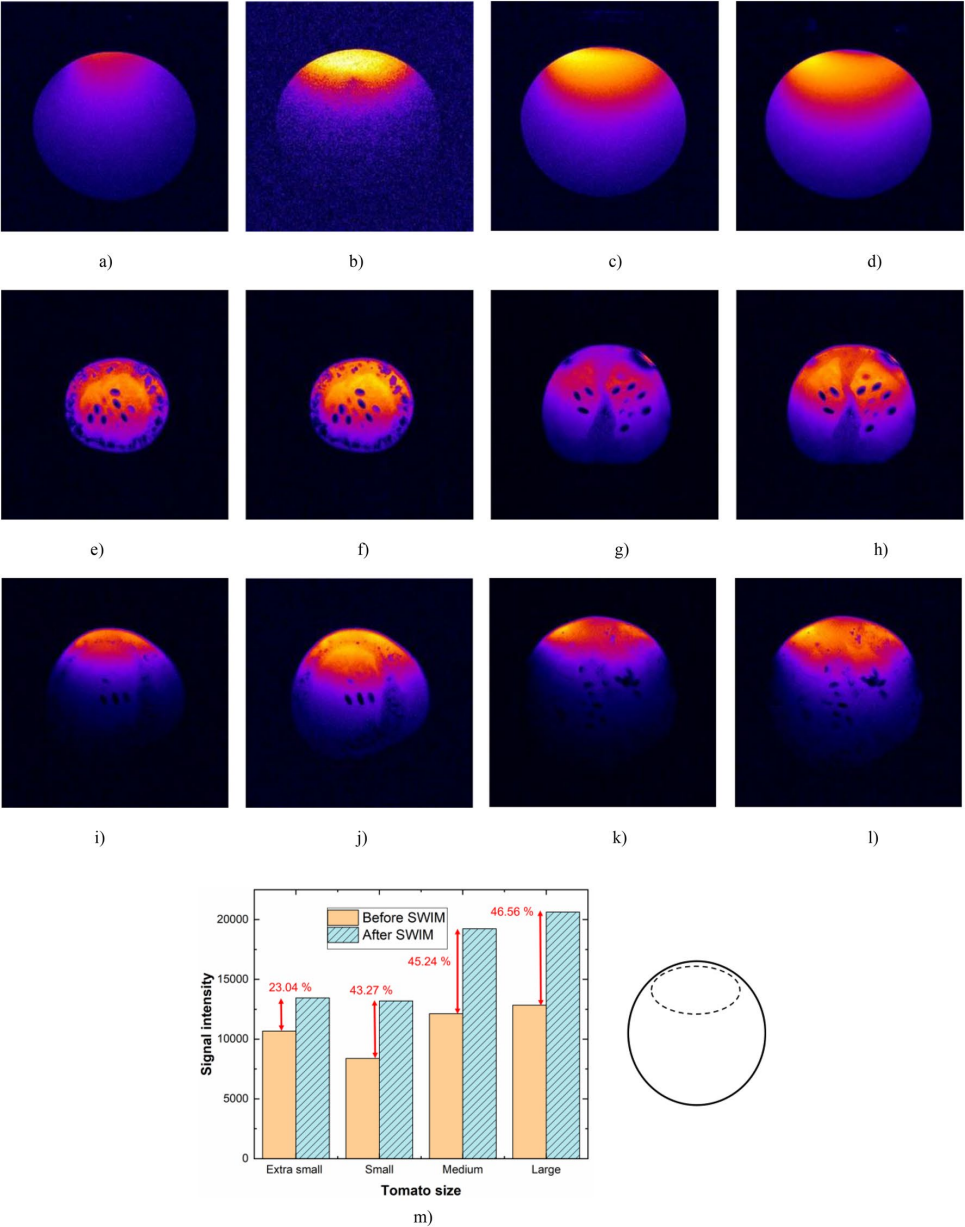
MR images of saline phantom, (**a**) mismatched condition after loading, (**b**) automatic matched condition by the SWIM system with active microcontroller and wireless communication, (**c**) automatic matched condition by the SWIM system with microcontroller in deep-sleep mode, (**d**) manually matched condition without the SWIM system. MR images of various tomatoes before and after SWIM calibration, extra small tomato (**e**,**f**), small tomato (**g**,**h**), medium tomato (**i**,**j**), and large tomato (**k**,**l**). (**m**) Signal intensity plot of different tomatoes before and after SWIM, calculated from the region closest to the coil as shown beside.

After loading the saline sample, retuning of the coil was not performed, and an image was acquired to show the effect of impedance mismatch. Two studies were performed to analyze the noise of the proposed system, with microcontroller in deep sleep mode and with microcontroller set to active mode. SNR of the ROI for the mismatched impedance condition was measured as 26.85 dB shown in Fig. 8a. However, when the microcontroller was in active mode with continuous wireless communication, the noise levels were significantly higher causing a drop in SNR from 26.85 to 24.08 dB (10.8% decrease) even after automatic impedance matching as shown in Fig. 8b. We then proceeded to place the controller in deep sleep mode where all the SWIM system processes are electrically switched off and the optimal impedance condition was set to the MEMS switches. This has shown substantial improvement in SNR from 26.85 to 34.15 dB as shown in Fig. 8c. To fully characterize the effectiveness of the SWIM system we also performed manual tuning and matching using trimmer capacitors without SWIM system and computed the SNR to be 34.96 dB as shown in Fig. 8d.

Four different cherry tomatoes were imaged to validate the general-purpose performance of the SWIM system. Before and after SWIM calibration images of extra small tomato (d = 21 mm), small tomato (d = 23 mm), medium tomato (d = 26 mm), and large tomato (d = 29 mm) are shown in Fig. 8e–l respectively. As tomatoes contain many inhomogeneities such as air bubbles, water, and seeds, accurately calculating the SNR is a challenge. Therefore, we present the mean signal levels from the ROI for each tomato (before and after SWIM) in Fig. 8m to show the impact of the SWIM system. As a surface coil was used for imaging, an ROI was identified close to the coil as marked with dashed circle in Fig. 8m (inset) for all the above calculations. The mean signal intensity has improved by 23.04% for extra small tomato, 43.27% for small tomato, 45.26% for medium tomato, and 46.56% for large tomato respectively. The larger the mismatch caused by the sample, the larger is the improvement in mean signal intensity as it is retuned to center frequency after the SWIM calibration sequence.

## Discussion

Two cases of remote matching setup were analyzed in ADS (1) with both capacitors C_M_ and C_M1_ (2) without C_M_ at the coil end. Coil design with only C_T_ showed more control over the tuning compared to the system setup shown in Fig. 5a. Nonetheless, during bench tests, both topologies showed adequate tuning and matching range to compensate for various phantoms representing small animals like mouse and rat for the chosen coil diameter. For coils with larger diameter, removing matching capacitor C_M_ at the coil end increased the frequency range of tuning. We concluded that the user could choose between the two options based on coil diameter and number of distributive capacitors. We used a brute force technique to find the minimum reflected power for a given sample. We integrated a bubble sort algorithm within the impedance state sweep to generate a time-efficient routine compared to a look-up-table method. ADC values were averaged to avoid erroneous power detector readings. Since the intention was to develop a general-purpose system meant to tune and match almost any small animal receive coil at 300 MHz, brute force routines work quickly as long as there are a small number of impedance states. Advanced optimization techniques like a gradient descent algorithm can be implemented to use the input PRESET state as a basis to find the lowest reflected power for a particular receive coil setup or an array of receive coils, by reducing time taken to match individual coils using the PRESET condition.

The MR images show that retuning and matching the coil after loading the sample will considerably increase the SNR of the coil. We calculated peripheral and overall SNR of the saline sample images (as saline sample is homogeneous) to fully characterize the improvement caused by the SWIM system. The SNR of the full image area increases by 17.2% (22.6–26.85 dB) after retuning the coil with the SWIM system. We also observed an increase in SNR at the bottom of the sample (opposite side of the coil) by 19.35% from 14 to 17 dB after the automatic calibration. The top side has seen an increase of 24% (26.85–34.15 dB) as the coil is closely placed on the sample. We also computed the SNR from left and right side of the image which showed 14.2% (18.06–20.82 dB) improvement after using the SWIM system. An image of the same sample with similar conditions was acquired with manual impedance matching using trimmer capacitors, where the SNR was calculated to be 34.96 dB. This shows a 2.34% decrease in SNR when compared to the image acquired using the SWIM system. A 1.2 dB insertion loss from SWIM components was measured during the bench tests, which include RF SPDT switch, bidirectional coupler, and the matching network with MEMS capacitor array. Along with the insertion loss of the system, microcontroller in active state induces noise further degrading the SNR even after an optimal impedance condition has been achieved by the SWIM system. Therefore, we used the ESP32’s deep sleep protocol after calibration to electrically power off the processing unit, RAM and all the digital peripherals except for RTC timer to wake the system for subsequent adjustments or power monitoring. RF MEMS switches retain the last received state via SPI and help maintain the optimal impedance condition during the deep sleep mode. Deep sleep acts as a power saving function as the controller consumes only 10 µA current in deep sleep compared to ~ 120 mA during the active state with continuous wireless communication. Various conditions were explored to offer a good starting position for the system but, we concluded that an option to decide the PRESET condition must be given to the user to customize the SWIM system for their needs. The system takes around 3 s to complete the process of tuning and matching with a brute force algorithm. The SWIM system provides the user with a greater level of monitoring and control than existing systems. Preclinical systems present a challenge for manual tuning with long tuning sticks and narrow bore sizes. Wireless communication combined with speed and efficiency enables effortless operation of the complete standalone system to avoid long cables and tuning sticks in preclinical scanners.

## Conclusion

A general purpose, fully standalone wireless system for impedance matching of receive RF coils was designed and evaluated at 7 T. This system was designed to compensate for the loading effect caused by various sample sizes by performing calibration using an RF CW signal generated on board. A Bluetooth enabled controller uses reflected power as feedback to perform the automatic tuning and matching. An android based mobile application was developed to operate the automatic and pseudo-manual controls of the system and monitor the reflected power in real-time. The system has demonstrated calibration within 3 s by scanning 256 combinations using the MEMS switch capacitor array. We have also demonstrated software detuning with the help of RF gating signal as an input to the system. This low power system has an easy-to-use design and can be scaled to multiple channels effortlessly. Four different sized tomatoes along with saline sample are imaged with the SWIM system to compensate for the impedance mismatched caused by the samples. Retuning the coil after loading the sample has shown a 24% increase in the SNR (dB) of the saline sample with the SWIM system. The mean signal intensity of the MR images improved by 23.04% for extra small tomato, 43.27% for small tomato, 45.24% for medium tomato, and 46.56% for large tomato respectively. Although reducing the SNR of saline sample by 2.34% compared to a manually tuned coil, the SWIM system brings speed, efficiency, and convenience to the table.

## Data Availability

The datasets generated during and/or analyzed during the current study are not publicly available as this is an ongoing project with software and hardware updates for further studies but are available from the corresponding author on reasonable request.

## References

[1] Bloch F, Hanse WW (1946). The nuclear experiment. Phys. Rev..

[2] Vaughan JT, Griffiths JR (2012). RF Coils for MRI.

[3] Ugurbil K, Garwood M, Ellermann J, Hendrich K, Hinke R, Hu X, Kim SG, Menon R, Merkle H, Ogawa S (1993). Imaging at high magnetic fields: Initial experiences at 4T. Magn. Reson. Q.

[4] Vaughan JT, Garwood M, Collins CM, Liu W, DelaBarre L, Adriany G, Andersen P, Merkle H, Goebel R, Smith MB, Ugurbil K (2001). 7T vs. 4T: RF power, homogeneity, signal-to-noise comparison in head images. Magn. Reson. Med..

[5] Shajan G, Hoffmann J, Budde J, Adriany G, Ugurbil K, Pohmann R (2011). Design and evaluation of an RF front-end for 9.4 T human MRI. Magn. Reson. Med..

[6] Ugurbil K (2014). Magnetic resonance imaging at ultrahigh fields. IEEE Trans. Biomed. Eng..

[7] Vaughan JT, DelaBarre L, Snyder C, Tian J, Akgun C, Shrivastava D, Liu W, Olson C, Adriany G, Strupp J, Andersen P, Gopinath A, van de Moortele P-F, Garwood M, Ugurbil K (2006). 9.4T human MRI: Preliminary results. Magn. Reson. Med..

[8] Larmor, J. LXIII. On the theory of the magnetic influence on spectra; and on the radiation from moving ions. *Phil. Mag.* **44**(271), 503–512 (1897).

[9] Gruber B, Froeling M, Leiner T, Klomp DWJ (2018). RF coils: A practical guide for nonphysicists. J. Magn. Reson. Imaging..

[10] Leifer MC (1997). Resonant modes of birdcage coil. J. Magn. Reason..

[11] Kim YC, Kim HD, Yun B-J, Ahmad SF (2020). A simple analytical solution for the designing of the birdcage RF coil used in NMR imaging applications. Appl. Sci..

[12] Vaughan JT, Adriany G, Snyder CJ, Tian J, Thiel T, Bolinger L, Liu H, DelaBarre L, Ugurbil K (2004). Efficient high-frequency body coil for high-field MRI. Magn. Reson. Med..

[13] Sohn, S.-M., DelaBarre, L., Vaughan, J. T., & Gopinath, A. 8-channel RF head coil of MRI with automatic tuning and matching. in *Proc. IEEE MTT-S Int. Microwave Symp. Dig.* (2013).

[14] Dubois M, Vergara Gomez TS, Jouvaud C (2019). Enhancing surface coil sensitivity volume with hybridized electric dipoles at 17.2 T. J. Magn. Reson..

[15] Hoult D (1978). The NMR receiver: A description and analysis of design. Prog. Nucl. Magn. Reson. Spectrosc..

[16] Sankey, L., & Popovic, Z. Adaptive tuning for handheld transmitters. in *Proc. IEEE MTT-S Int. Microw. Symp. Dig.*, pp. 225–228 (2009).

[17] De Mingo J, Valdovinos A, Crespo A, Navarro D, Garcia P (2004). An RF electronically controlled impedance tuning network design and its application to an antenna input impedance automatic matching system. IEEE Trans. Microw. Theory Tech..

[18] Madic, J., Bretchko, P., Shuyun, Z., Shumovich, R., & McMorrow, R. Accurate power control technique for handset PA modules with integrated directional couplers. in *Proc. Radio Frequency Integrated Circuits Symp.*, pp. 715–718 (2003).

[19] Song H, Aberle JT, Bakkaloglu B (2010). A mixed-signal matching state search based adaptive antenna tuning IC. IEEE Microwave Wirel. Compon. Lett..

[20] Sjoblom P, Sjoland H (2005). An adaptive impedance tuning CMOS circuit for ISM 2.4-GHz band. IEEE Trans. Circuits Syst. I Regul. Pap..

[21] Seigneuret, G., Bergeret, E., & Pannier, P. Auto-tuning in passive UHF RFID tags. in *Proceedings of the 8th IEEE International NEWCAS Conference 2010*, pp. 181–184 (2010). 10.1109/NEWCAS.2010.5603749.

[22] Wegleiter H, Schweighofer B, Deinhammer C, Holler G, Fulmek P (2011). Automatic antenna tuning unit to improve RFID system performance. IEEE Trans. Instrum. Meas..

[23] Mohan A, Mondal S (2021). An impedance matching strategy for micro-scale RF energy harvesting systems. IEEE Trans. Circ. Syst. II Express Briefs.

[24] Zhao, P., Zheng, Y., & Glesner, M. Automatic impedance matching in Microwave power harvesters. In *6th Conference on Ph.D. Research in Microelectronics & Electronics*, pp. 1–4 (2010).

[25] Hwang F, Hoult DI (1998). Automatic probe tuning and matching. Magn. Reson. Med..

[26] Hirata H, Yamaguchi Y, Takahashi T, Luo ZW (2003). Control characteristics of an automatic matching control system for in vivo EPR spectroscopy. Magn. Reson. Med..

[27] Pérez de Alejo R, Garrido C, Villa P, Rodriguez I, Vaquero JJ, Ruiz-Cabello J, Cortijo M (2004). Automatic tuning and matching of a small multifrequency saddle coil at 4.7 T. Magn. Reson. Med..

[28] Muftuler LT, Gulsen G, Sezen KD, Nalcioglu O (2002). Automatic tuned MRI RF coil for multinuclear imaging of small animals at 3T. J. Magn. Reson..

[29] Sohn S-M, DelaBarre L, Gopinath A, Vaughan JT (2015). Design of an electrically automated RF transceiver head coil in MRI. IEEE Trans. Biomed. Circuits Syst..

[30] Mehmann A, Vogt C, Varga M, Port A, Reber J, Marjanovic J, Pruessmann KP, Sporrer B, Huang Q, Troster G (2019). Automatic resonance frequency retuning of stretchable liquid metal receive coil for magnetic resonance imaging. IEEE Trans. Med. Imaging.

[31] Jouda M, Torres Delgado SM, Jouzdani MA, Mager D, Korvink JG (2020). ArduiTaM: accurate and inexpensive NMR auto tune and match system. Magn. Reson..

[32] Venook RD, Hargreaves BA, Gold GE, Conolly SM, Scott GC (2005). Automatic tuning of flexible interventional RF receiver coils. Magn. Reson. Med..

[33] Pavan, M., & A. K. P. P. A modular automatic matching network system. in *Proc. 18th Int. Soc. Magnetic Resonance in Medicine Meeting*, p. 647 (2010).

[34] Wu, S., Beck, B. L., Turner, W. J., Bashirullah, R., & Mareci, T. An automatic impedance matching system for multiple frequency coils. In *Proc. ISMRM*, July, 3920 (2010).

[35] Beck, B. L., Turner, S. W. W. J., Bashirullah, R., & Mareci, T. H. High Q reactive network for automatic impedance matching. in *Proc. 19th Int. Soc. Magnetic Resonance in Medicine Meeting*, p. 1853 (2011).

[36] Kandala, S.K., & Sohn, S. Wirelessly controlled stand-alone automatic RF tuning and matching system for preclinical imaging at 7T.in *Proc. 30th Int. Soc. Magn. Resonance Med.*, p. 2515 (2021).

[37] Twieg M, de Rooij MA, Griswold MA (2015). Active detuning of MRI receive coils with GaN FETs. IEEE Trans. Microw. Theory Tech..

[38] Saha S, Pricci R, Koutsoupidou M (2020). A smart switching system to enable automatic tuning and detuning of metamaterial resonators in MRI scans. Sci. Rep..

[39] Mitchell MD, Kundel HL, Axel L, Joseph PM (1986). Agarose as a tissue equivalent phantom material for NMR imaging. Magn. Reson. Imaging..

[40] Kodibagkar VD, Conradi MS (2000). Remote tuning of NMR probe circuits. J. Magn. Reson..

[41] Pesel RG, Attar SS, Mansour RR (2015). MEMS-based switched-capacitor banks for impedance matching networks. Eur. Microw. Conf. (EuMC).

[42] Pozar DM (2012). Microwave engineering.

[43] Goerner FL, Clarke GD (2011). Measuring signal-to-noise ratio in partially parallel imaging MRI. Med. Phys..

